# Macroevolutionary diversification with limited niche disparity in a species-rich lineage of cold-climate lizards

**DOI:** 10.1186/s12862-018-1133-1

**Published:** 2018-02-06

**Authors:** Ashley M. Reaney, Mónica Saldarriaga-Córdoba, Daniel Pincheira-Donoso

**Affiliations:** 1 Laboratory of Evolutionary Ecology of Adaptations, School of Life Sciences, University of Lincoln, Brayford Campus, Lincoln, Lincolnshire LN6 7DL UK; 2 Centro de Investigación en Recursos Naturales y Sustentabilidad, Universidad Bernardo O’Higgins, Santiago, Chile; 3 Department of Life Sciences, Imperial College London, Silwood Park Campus, Buckhurst Road, Ascot, Berkshire, SL5 7PY UK

**Keywords:** Natural selection, Non-adaptive radiation, Diversification, Niche conservatism, Macroevolution, Lizards, *Phymaturus*

## Abstract

**Background:**

Life diversifies via adaptive radiation when natural selection drives the evolution of ecologically distinct species mediated by their access to novel niche space, or via non-adaptive radiation when new species diversify while retaining ancestral niches. However, while cases of adaptive radiation are widely documented, examples of non-adaptively radiating lineages remain rarely observed. A prolific cold-climate lizard radiation from South America (*Phymaturus*), sister to a hyper-diverse adaptive radiation (*Liolaemus*), has extensively diversified phylogenetically and geographically, but with exceptionally minimal ecological and life-history diversification. This lineage, therefore, may offer unique opportunities to investigate the non-adaptive basis of diversification, and in combination with *Liolaemus*, to cover the whole spectrum of modes of diversification predicted by theory, from adaptive to non-adaptive. Using phylogenetic macroevolutionary modelling performed on a newly created 58-species molecular tree, we establish the tempo and mode of diversification in the *Phymaturus* radiation.

**Results:**

Lineage accumulation in *Phymaturus* opposes a density-dependent (or ‘niche-filling’) process of diversification. Concurrently, we found that body size diversification is better described by an Ornstein-Uhlenbeck evolutionary model, suggesting stabilizing selection as the mechanism underlying niche conservatism (i.e., maintaining two fundamental size peaks), and which has predominantly evolved around two major adaptive peaks on a ‘Simpsonian’ adaptive landscape.

**Conclusions:**

Lineage diversification of the *Phymaturus* genus does not conform to an adaptive radiation, as it is characterised by a constant rate of species accumulation during the clade’s history. Their strict habitat requirements (rocky outcrops), predominantly invariant herbivory, and especially the constant viviparous reproduction across species have likely limited their opportunities for adaptive diversifications throughout novel environments. This mode of diversification contrasts dramatically with its sister lineage *Liolaemus*, which geographically overlaps with *Phymaturus*, but exploits all possible microhabitats in these and other bioclimatic areas. Our study contributes importantly to consolidate these lizards (liolaemids) as promising model systems to investigate the entire spectrum of modes of species formations, from the adaptive to the non-adaptive extremes of the continuum.

**Electronic supplementary material:**

The online version of this article (10.1186/s12862-018-1133-1) contains supplementary material, which is available to authorized users.

## Background

The diversification of life via evolutionary radiations is mediated by multiple modes of lineage proliferation that range from the adaptive to the non-adaptive extremes of the speciation continuum [1, 2]. On one extreme, adaptive radiations occur when a lineage diversifies into ecologically different species through divergent natural selection [1, 2] – a process that leads to clades displaying exceptional ecological and morphological diversity [3]. Ecological opportunity is a fundamental requirement underlying this process [1, 4], which offers clades an array of available resources that promote the proliferation of new species via divergent adaptation to alternative regions of the niche spectrum via ecological specialization [1, 5, 6]. The predominant role of adaptive radiation theory has been reinforced by the emergence of multiple empirical examples from across multiple lineages, including *Anolis* lizards [7, 8], Darwin’s finches [9], African cichlids [10], and more recently, *Liolaemus* lizards [11, 12]. On the other extreme, lineages have been observed to proliferate displaying minimal or no ecological diversification, and hence, under a process that does not involve divergent natural selection driving new species to specialize in alternative areas of the niche spectrum (i.e., no niche-filling is involved) [2, 13]. This process, known as non-adaptive radiation, predicts that spatial isolation among newly evolving species can facilitate the evolution of reproductive isolation with niche conservatism, thus resulting in diversifying lineages whereby species occupy similar niches in non-overlapping areas [2]. Examples of taxa having undergone non-adaptive diversifications include the salamander genera *Batrachoseps* [14] and *Plethodon* [15], and invertebrate lineages such as *Albinaria* [13] and *Achatinella* [16] snails. In contrast with clades diversifying via adaptive radiation, empirical cases of non-adaptive radiations are considerably more infrequent and have received less attention. How widespread non-adaptive radiations are in nature and whether factors such as proliferation in archipelago or mainland backgrounds plays an effect on their likelihood of expressing remain open questions.

An peculiar evolutionary scenario is observed in South America within the family Liolaemidae, where two species-rich sister genera of geographically overlapping lizards have radiated across a range of climates that cover some of the planet’s coldest environments occupied by reptiles. These clades, *Liolaemus* and *Phymaturus*, display highly contrasting patterns of biodiversity distribution. At one extreme, *Liolaemus* is one of the most prolific genera among living vertebrates, numbering 270+ species [17] and inhabiting one the widest ranges of environmental/climatic conditions recorded among reptiles [12, 18–20]. These unique features have contributed to consolidate *Liolaemus* as a highly promising vertebrate model system to investigate diversification, adaptation and extinction theories [21–24]. For example, while the overwhelming majority of research on adaptive radiation has concentrated on clades distributed on ‘island’ systems (e.g. archipelagos, lakes), leading to the idea that such phenomenon is unlikely to take place in mainland settings [25], recent studies suggest that the *Liolaemus* prolific diversification is consistent with an adaptive radiation [11, 12]. In striking contrast, its sister clade *Phymaturus* has actively diversified into 60+ species that exhibit minimal or no phenotypic and ecological differentiation. All known *Phymaturus* are specialized in the use of rocky microhabitats, have (almost entirely) herbivorous diets, viviparous (live-bearing) reproduction, and are characterized by a robust and flattened body shape and predominantly distinctive sexual dimorphism both in size and colouration [26–35]. *Phymaturus* has diversified into two major clades, one Patagonian (*patagonicus* clade) and one Andean (*palluma* clade), yet, both subgroups have been suggested to have undergone the same mode of lineage diversification [33, 34, 36]. This propensity to conserve major components of their ancestral niche [30] is associated with a highly structured spatial pattern characterized by a tendency for species to occupy small geographic ranges isolated from each other [30, 33, 35]. Therefore, coexistence among *Phymaturus* species is extremely unusual [37, 38]. Collectively, the combination of these ecological, evolutionary and geographic patterns has promoted the view that *Phymaturus* lizards have diversified via non-adaptive radiation [33]. Therefore, this prolific lizard family has been predicted to hide an immense component of non-adaptive diversification (in *Phymaturus*) that has remained eclipsed by the exceptional evolutionary history of *Liolaemus*.

Here, we employ a model-selection approach to investigate the tempo and mode of evolutionary diversification of the *Phymaturus* non-adaptive radiation. Our study aims to address the hypothesis that the diversification of the *Phymaturus* genus deviates from a density-dependent mode of evolution and therefore, we do not expect to find evidence that a ‘niche-filling’ mode of species accumulation has led to the consistently observed conservatism of niche occupation in this clade. That is, we expect an early evolutionary history characterized by slow diversification that accelerates over time arithmetically as the accumulation of more ancestors (nodes in the phylogeny) circumstantially allows the origins of more new species. Examination into the evolutionary mode of *Phymaturus* will ascertain whether this clade exemplifies a non-adaptive radiation providing i) a unique insight into the phenomenon and enhancing our understanding of the full spectrum of evolutionary radiations, from adaptive (i.e. niche-filling mediated) to non-adaptive (no niche-filling) and ii) how disparate modes may be expressed within one single lineage (Liolaemidae family). We will employ a range of phylogenetically based modelling analyses to quantitatively establish the most likely model describing the diversification history that has given origin to this intriguing genus of cold-climate lizards.

## Methods

### Phylogeny and divergence time analysis

We employed a Bayesian relaxed molecular clock method with uncorrelated lognormal rates among branches [39], assuming a Yule tree prior to the speciation model as implemented in Beast v.1.8.0 [40] to estimate divergence dates. We then time-calibrated the inferred phylogenetic tree based on 19 species of *Liolaemus* by estimating the age of divergence of the node that led to the *Eulaemus*-*Liolaemus* (sensu stricto) divergence, based on the *Liolaemus* fossil and geological record with lognormal distributions [11, 41]. The calibration point was set to the minimum age for the genus suggested by Albino [41] of ~ 20 to 18.5 million years since the present (Mya). This is estimated from remains of the *Liolaemus* genus from the Early Miocene of Gaiman (Sarmiento Formation, Chubut, Argentina) according to the calibration of the Colhuehuapian Age proposed by Madden [42]. The complete dataset used to perform this analysis was carried out with the aforementioned 19 *Liolaemus* species along with 58 species of *Phymaturus* (see accession numbers in Additional File 1, and phylogenetic tree in NEXUS in Additional File 2). The concatenated matrix was performed with three gene sequences for each individual, two mitochondrial genes (Cytochrome b and 12S ribosomal RNA), and one nuclear gene (c-mos), each downloaded from the GenBank database (http://www.ncbi.nlm.nih.gov/genbank). The alignment of each dataset was performed in BioEdit version 7.0 [43] and confirmed by eye using GeneDoc [44]. Following Triant & DeWoody [45], Cyt b and c-mos sequences were translated into amino acids to check for premature stop codons or other nonsense mutations, which would have indicated the amplification of nuclear mitochondrial translocations (numts). Best-fit models of evolution were estimated for each dataset using MrModeltest [46] and chosen according to their Akaike Information Criteria (AIC) following Bos & Posada [47]. The model selected was GTR + I+Γ for the mitochondrial genes and HKY+Γ model for the nuclear gene (see phylogeny with node support in Additional File 3).

Analyses were run for 60 million generations, with samples retained every 1000 generations. Results were displayed in Tracer to confirm acceptable mixing and likelihood stationarity of the Markov chain Monte Carlo (MCMC) analyses, appropriate burn-in and adequate effective sample sizes (> 200) for each estimated parameter. We summarized parameter values of the samples from the posterior on the maximum clade credibility tree using TreeAnnotator 1.8.0 with the posterior probability limit set to 0.5 and mean node heights summarized. We used a lognormal prior for the treeModel.root height parameter, and the following additional constraints according to Breitman et al. [48]. The stem of the *Eulaemus* origin was constrained with a zero offset (hard upper bound) of 18.5 Ma, a lognormal mean of 1.0, and a lognormal standard deviation of 1.5. This produced a median age centred at 21.22 Ma and a 95% prior credible interval (PCI) at 50.55 Ma.

### Analyses of lineage diversification

Analyses based on this new time-calibrated phylogenetic tree, with a focus on both lineage and body size diversity (see next section), were performed to quantify the tempo and mode of *Phymaturus* diversification. To calculate historical rates of species accumulations we created a lineage through-time (LTT) plot implemented in the R package ‘ape’ [49]. Pybus & Harvey’s [50] Monte Carlo Constant Rate (MCCR) test was first implemented to create the LTT plot. This analysis calculates the *γ* statistic for incompletely sampled phylogenies, by comparing the distribution of inter-node distances between the tree root and its temporal midpoint to that of the temporal midpoint and the tips of the tree [51]. Negative values indicate that inter-node distances between the root and midpoint are shorter in comparison to the distances between the midpoint and tree tips, while positive values indicate the opposing trend. In the former case, most branching events occur earlier in the clade’s evolutionary history, and therefore, this pattern is considered to be consistent with an ‘early-burst’ of diversification which describes a decline in the rate of species accumulation over time. When lineage diversification is described by a constant rate, branching events are evenly distributed throughout the tree and *γ* is normally distributed with a mean of 0. Given that type I error rates increase among incompletely sampled phylogenies, the MCCR test accounts for such errors by calculating corrected *γ* distributions through many simulations of the known clade size (in this case, assumed to be 65 species of *Phymaturus*) under the null hypothesis of a constant rate pure-birth diversification process. Species are subsequently removed randomly from the simulated trees to replicate incomplete sampling. Our analysis is based on 10,000 Monte Carlo simulations conducted using the ‘laser’ package in R [52]. Subsequently, we investigated the diversification dynamics that are more likely to have sculpted the LTT trend in *Phymaturus* by fitting multiple evolutionary models that represent alternative evolutionary dynamics of lineage accumulation. We tested four alternative hypotheses describing modes of diversification using Etienne et al.’s [53] maximum-likelihood fitting model method, which utilises a hidden Markov model (HMM) approach to calculate the probability of multiple models on the phylogenetic history of our model clade. Etienne et al.’s method is particularly appropriate as it runs models which account for the influence that absent species (both extinct and missing from the phylogeny) may have on historical rates of diversification. As such, this methodology is equivalent to the MCCR test output above as both techniques consider the potential effects of missing species from the phylogeny [51]. Two of the four fitted models, the pure-birth (or Yule) model and constant rate birth-death model (crBD), assume constant diversification rates. While the former assumes no extinctions, the latter does, but assumes that the rates of speciation and extinction remain constant throughout time across the lineages. Both the other two models implemented in Etienne et al.’s approach, density-dependent logistic (DDL + E) and density-dependent exponential (DDE + E), assume diversity-dependence and hence quantify diversification rates over time as functions of changes in accumulating diversity while simultaneously accounting for extinctions (E). The DDL + E models diversification changes linearly whereas the DDE + E models speciation declining exponentially as a function of extant lineage diversity at any point in time. All four models were fitted under three alternative assumptions about the ‘actual’ diversity of the *Phymaturus* clade (and the proportion of missing species in the phylogeny): (1) the assumption that the *Phymaturus* genus consists of its currently known 65 species (however, our phylogeny contains 58 of the 65 known species [35]), (2) that it consists of 70 species (of which 58 are sampled), and (3) that 80 species of *Phymaturus* exist (of which 58 are sampled). These assumptions are aimed to establish whether selection of evolutionary models is sensitive to proportion of missing species in the available phylogeny – consistency in model-selection would reinforce conclusion of identification of the process that best describes the diversification of this lineage over time [11, 53]. For all three assumptions, all four models were fitted using the R package ‘DDD’ [53]. Evaluation of the best-fit model was achieved by employing the Akaike Information Criterion (AIC) approach [54] reporting the bias-corrected version of AIC (referred to as AICc [55, 56]). By identification of the lowest AICc the most appropriate candidate evolutionary models can be determined, hence, when presented as ΔAICc scores (between the highest and lowest AICc of each model), the best model has a ΔAICc = 0 [55, 56].

### Body size data

We investigated the evolutionary trajectories and rates of body size diversification in *Phymaturus* to establish relationships between diversification of lineages and of a phenotype with a primary ecological, physiological and life-history role, and thus, with a prime role in evolutionary processes [57, 58]. Body size is generally considered to offer a key proxy for niche across natural populations [57, 59]. We measured snout-vent length (SVL) as this variable provides the traditional proxy for body size in lizards [60–62]. For these analyses, we collated an extensive body size dataset consisting of 1200+ adult individuals (specimens were categorized as sexually mature after analysing the presence of mature gonads and the functional development of secondary sex characters, [33]) spanning 41 out of the 58 species included in our phylogeny (Additional File 4). To obtain SVL for each species, we averaged male and female SVL values, calculated independently using the upper two-thirds of the size range available for each sex in each species [63, 64]. We chose this approach over the extensively used methods based on maximum SVL as a proxy for size in lizards, because it has been shown that the use of extreme values may lead to overestimations of body size [61], while the use of intermediate percentiles between the maximum recorded value and the mean from the entire adult sample provides more accurate estimates of asymptotic size [65]. The species included in our dataset encompass the entire phylogenetic, phenotypic, ecological, and geographic diversity known within the *Phymaturus* genus, and therefore, they provide an adequate sample of the body size diversity in this genus.

### Modelling body size evolution

We first modelled body size disparity through time (DTT) based on body size data from extant species (see above). The DTT method, conducted using the R package ‘geiger’ [66], calculates mean trait relative disparity and compares observed body size disparity to that expected under the null model of Brownian-motion through 10,000 simulations of body size evolution across the tree [67]. Average body size disparity was then obtained from both the real and simulated data and plotted against node age to calculate the morphological disparity index (MDI). This MDI quantifies the overall difference in relative disparity for body size both among and within subclades compared with that expected under Brownian motion [66, 68, 69]. A lower than expected trait disparity under Brownian motion would produce a negative MDI value (i.e., low average subclade relative disparity), meaning most disparity occurs among subclades, thus occupying minor, isolated areas of the morphospace [67]. Contrastingly, should relative disparity among subclades show a stronger overlap in the morphospace a positive MDI value will be returned [67]. Using the ‘contMap’ and ‘FancyTree’ functions in the ‘phytools’ package in R [70], the *Phymaturus* phylogeny was projected onto the body size morphospace (against time since root), based on ancestral node estimations using maximum likelihood [71].

We then employed two quantitative approaches to identify the model underlying the evolutionary dynamics of body size throughout the history of *Phymaturus*. Firstly, we fitted four alternative models each describing contrasting evolutionary dynamics to quantify the tempo and mode of body size diversification. The Brownian-motion model (BM) describes a ‘random walk’ of trait evolution with the variance of the trait proportional to time and centred around the initial value (at the tree’s root) [72]. The Ornstein-Uhlenbeck model (OU, which assumes that adaptively evolved traits are pulled by stabilizing selection around a fitness optimum; [73]), the Early- Burst or “niche-filling” model (EB, which describes exponential rates of evolution over time assuming niches are saturated by new and incipient species within a lineage; [74]), and the Delta model (detects whether recent trait evolution has accelerated or slowed over time; returning a δ > 1 value when recent evolution has been fast, or slow when δ < 1 [75]). Comparisons of goodness of fit were performed using the Akaike Information Criterion (AIC) [54] and the same AICc approach described for model-selection of lineage accumulation, conducted with the R package ‘geiger’ [66], was used to select the best evolutionary model (see above). We then employed the ‘surface’ package in R [76, 77] to investigate whether body size distribution among *Phymaturus* species has evolved around one or multiple SVL optima (i.e., if stabilizing selection has produced convergences of body size on a macroevolutionary scale creating one or more peaks within the genus). This process builds on the ‘OUCH’ method [73] by fitting OU stabilizing selection models in which lineages may display convergent shifts towards adaptive optima on a Simpsonian landscape (i.e., a theoretical landscape upon which organism phenotypes are distributed spatially with height at any given location indicating the fitness of that particular phenotype). One advantage of this method is that it lacks any assumptions of whether certain lineages correspond to specific optima [76, 77]. ‘Surface’ employs a stepwise AICc model selection approach, permitting the identification of the best model along with the number and locations of the adaptive peaks (i.e., trait ‘regimes’), thus detecting convergence towards such optima over time [76, 77].

## Results

### Diversification rates and evolutionary models

**Fig. 1:**
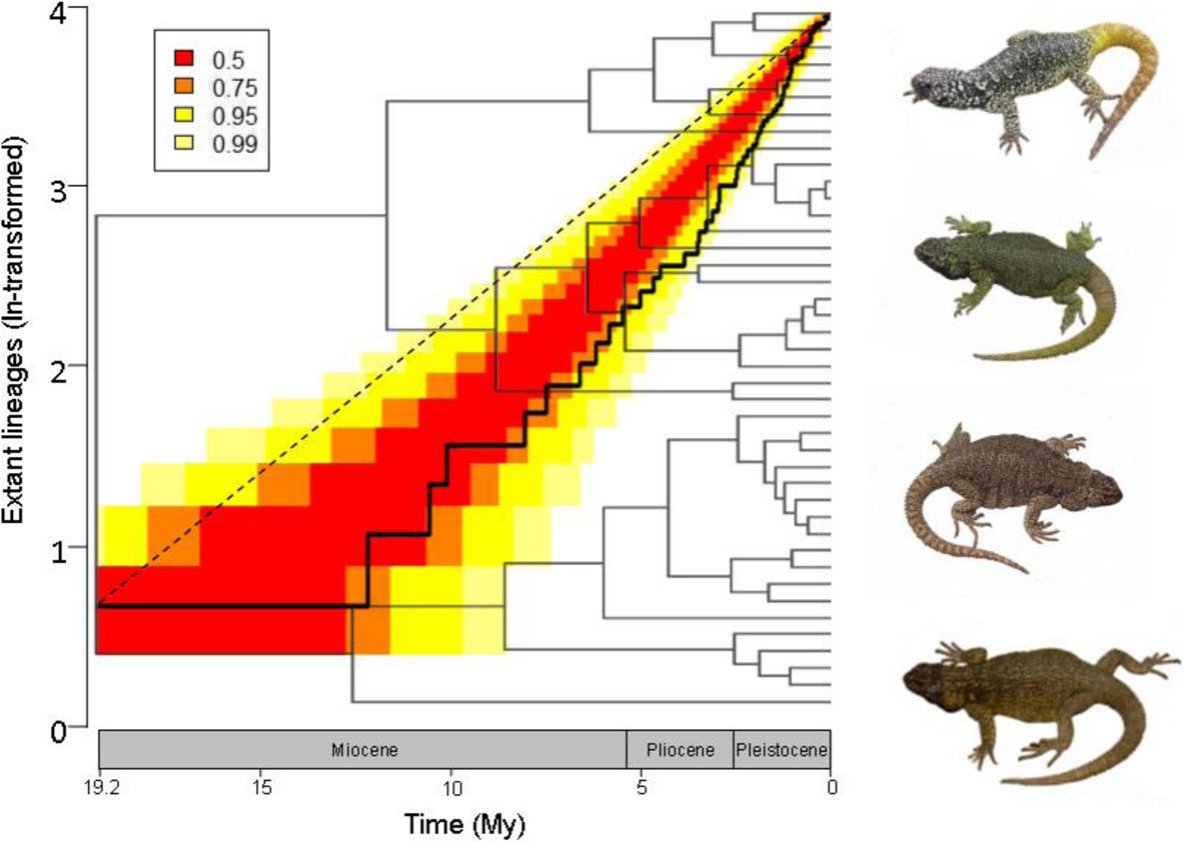
Tempo and mode of macroevolutionary diversification in *Phymaturus.* A lineage through-time (LTT) curve showing species accumulation over time (solid line) against that predicted under Brownian motion (dashed line). Confidence intervals are shown at 50% (red area) and 95% (yellow area). The phylogeny of *Phymaturus* is shown in the background of the LTT plot. Images of *Phymaturus* are from top to bottom: *P. vociferator* (male), *P. maulense* (male), *P. maulense* (female), *P. palluma* (male)

The MCCR analysis reveals a lineage diversification curve characterized by a rather constant rate of species accumulation over time (*γ* statistics = − 1.86), as shown by the lineage through time plot (Fig. 1). The accumulation of lineages during *Phymaturus* history shows some episodes of significantly steep declines (where the lineage-diversification line falls outside the 95% confidence interval). These events took place soon after the Miocene-Pliocene boundary and reach the Pleistocene (Fig. 1). The model-selection analyses (based on an actual ~ 90% of known species sampled) identified the constant rate birth-death model (crBD) as the best approximation describing the macroevolutionary dynamics of lineage accumulation within *Phymaturus* (Table 1). Subsequent simulations of scenarios assuming *Phymaturus* ‘actual’ diversities of 70 (and thus, assuming that 83% of the species were sampled) and 80 species (and thus, assuming that 73% of the species were sampled), ranked the same model as the best alternative, which reinforces that model-identification is not an artefact of proportions of missing species from the phylogeny. However, although the crBD model was consistently ranked as the best alternative, the ∆AICc values are also consistently < 1 relative to the Yule model, while the differences with both density-dependent models (DDL + E and DDE + E) are consistently above the 2 threshold in ∆AICc values between models (Table 1).

**Table 1:** Rates of species accumulation during *Phymaturus* diversification history based on multiple evolutionary models

Model	*λ*	*μ*	LogL	AICc	∆AICc
58/65 Known					
Yule	5.765772	0	38.69399	−75.31655	0.51701
crBD	8.021884	4.389454	40.02587	−75.83356	0
DDL + E	6.834053	0.083624	39.81339	−73.18234	2.65122
DDE + E	6.928767	0.3788968	39.8202	−73.19596	2.6376
58/70 known					
Yule	5.954685	0	38.40051	−74.72959	1.00081
crBD	8.524321	4.890441	39.97429	−75.7304	0
DDL + E	7.121737	0.083370	39.8481	−73.25176	2.47864
DDE + E	7.237836	0.390419	39.85628	−73.26812	2.46228
58/80 known					
Yule	6.223624	0	37.94997	−73.82851	1.75693
crBD	9.278077	5.642338	39.90181	−75.58544	0
DDL + E	7.955254	1.167757	39.92335	−73.40226	2.18318
DDE + E	7.64201	0.404501	39.9097	−73.37496	2.21048

Fitted models include pure-birth (Yule), birth-death (crBD), density-dependent logistic (DDL + E) and density-dependent exponential diversification (DDE + E). The best-fit of models are based on (delta) bias-corrected AIC (∆AICc)

### Tempo and mode of body size evolution

**Fig. 2:**
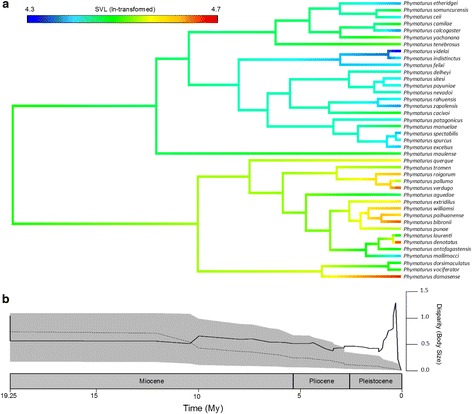
Disparity in body size among species of *Phymaturus.* (**a**) Maximum likelihood phylogenetic reconstruction of ancestral body sizes (ln-transformed) with the interspecific range shown in the coloured bar. (**b**) Mean subclade disparity through-time (DTT) for body size (solid line) compared with median subclade DTT under Brownian motion (dashed line). The grey area represents the 95% confidence interval of DTT range based on simulations of body size. Median subclade disparity was calculated based on 10,000 simulations of phenotypic evolution on the *Phymaturus* phylogeny

The results of the disparity through time (DTT) analysis, revealed a predominantly limited degree of relative body size disparity throughout the evolutionary history of the clade (MDI = 0.089), with a strong change in the diversification dynamics in this trait that was estimated to have taken place around 10Mya (Fig. 2). This analysis shows a curve characterized by a lower relative disparity than expected by chance during the first half of the *Phymaturus* history, when limited body size variation remained constrained within clades with no overlap in morphospace (Fig. 2). This period of limited diversification is found to have been followed by a steep increase in relative body size disparity that reveals a substantial increase accentuated over the last ~ 2.6My which substantially exceed the 95% confidence interval calculated by our 10,000 simulations (Fig. 2). A first diversification pulse occurred around the Pliocene-Pleistocene boundary, while a second pulse expressed prominently over the last million years of the clade history. The results of the contMap (Fig. 2) illustrate this finding further by depicting the most likely path that body size evolution has taken. The two subclades of *Phymaturus* are shown with species of the *patagonicus* clade (top subclade) having relatively smaller body sizes (tree tips are primarily blue/green) compared to species of the *palluma* clade (bottom subclade) which typically have larger body sizes, and importantly, show a considerably higher degree of evolutionary lability in this trait as evidenced by dynamic transitions along the tree branches. These changes strongly contrast with the degrees of change observed among species of the *patagonicus* clade (Fig. 2).

**Fig. 3:**
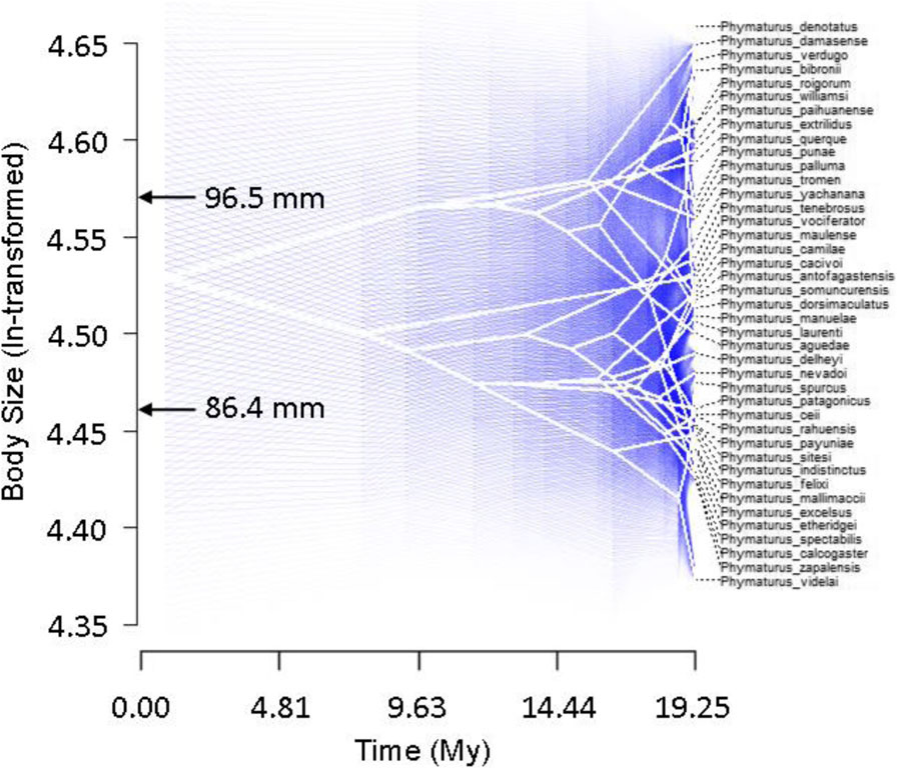
Projection of the *Phymaturus* phylogeny onto a morphospace of body size (ln-transformed) as a function of time (in million years elapsed since the root). The level of uncertainty is expressed as increasing blue transparency around the branches. Arrows indicate the position of the two body size peaks (in mm) identified using surface analysis

The model-based analysis identifies the Ornstein-Uhlenbeck (OU) model as the best approximation describing the evolutionary diversification of body size in *Phymaturus* (Table 2). This analysis indicates that body size has diversified subject to stabilising selection forcing its evolution around two adaptive peaks revealed by our surface-based analyses on a Simpsonian landscape (Fig. 3; Additional files 5 and 6). The first of these peaks encompasses the majority of the *patagonicus* clade at around 86.4 mm in SVL, while the second includes the majority of the *palluma* clade, characterized by larger species, at approximately 96.5 mm (Fig. 3; Additional Files 5 and 6). The remaining models were highly differentiated from the OU model by ∆AICc ranging from 11.3–20 (Table 2).

**Table 2:** Rates and modes of evolutionary diversification in *Phymaturus* body size based on comparisons of four evolutionary models

Model	Model Parameters	*β*	LogL	AICc	∆AICc
BM	–	0.001999	43.587975	−82.86016	17.7023
OU	*α* = 0.437549	0.004391	53.605606	−100.5625	0
EB	*α* = −0.000001	0.001999	43.587940	−80.5272	20.0353
Delta	*δ* = 2.999999	0.000727	47.942350	−89.236	11.3265

Fitted models are Brownian-motion (BM), Ornstein-Uhlenbeck (OU), Early-burst (EB) and Delta. Best fit models based on the (delta) bais-corrected Akaike Information Criteria (AICc)

## Discussion

Non-adaptive radiations are characterised by species formations associated with minimal ecological and phenotypic divergence [2, 13]. Our replicated analyses consistently rejected density-dependent models of lineage diversification across known and simulated species richness for the strictly cold-climate *Phymaturus* genus (Table 1). Therefore, our findings support the expectation that this lizard radiation has not diversified via formation of species mediated by niche-filling (Fig. 1), in strong contrast with its sister lineage *Liolaemus*, recognized as one of the world’s most prominent adaptive radiations [11]. These findings are paralleled by further analyses showing limited diversification of body size across species, characterised by only one major shift in body size plan (which led to the two major clades known within *Phymaturus*), and a more recent tendency for body size overlap in morphospace associated with allopatric distribution, which is thus likely to be facilitated by the lack of ecological interference among species of similar size. Collectively, therefore, our macroevolutionary findings identify the *Phymaturus* clade as an emerging example of a non-adaptive continental radiation, with the potential to open multiple unique and novel opportunities to (*i*) investigate the factors underlying the differing dynamics of evolutionary diversification seen in nature, but with the benefit of focusing on a scenario consisting of two contrasting, yet spatially overlapping and closely related lineages, (*ii*) consolidate the emerging view that the evolution of viviparous reproduction in reptiles can operate as a ‘double-edge sword’ trait by making radiations of lineages within extreme cold-climates possible [12], while imposing adaptive barriers to their radiation across warmer climates, where oviparous species predominate, and thus (*iii*) to highlight the role that such key adaptations can play in influencing the extent to which a clade diversifies as a result of their potential to expand across (or remain restricted to) certain climatic regions. Although *Liolaemus* and *Phymaturus* are sister lineages, the current diversity of the former is roughly four times greater than the latter, which could be a consequence of the ‘unbounded’ potential for *Liolaemus* to expand across environments, in contrast to *Phymaturus* which inhabit cold climates [27].

### Diversification of lineages

Lineage diversification within *Phymaturus* is described by a rather constant accumulation of species following a period of early slow diversification, a pattern consistent with a mode of non-adaptive radiation (Fig.1). This is contrasted with the opposing evolutionary trajectory recently shown in its sister clade *Liolaemus,* which follows a density-dependent (i.e., ‘niche-filling’) curve of lineage accumulation showing episodic pulses of diversification throughout their evolutionary history [11] combined with extraordinary ecological and morphological diversity [12, 18].

Recent studies have suggested an important role for viviparous reproduction (live-bearing parity mode, which acts as a maternal incubator for embryos) as a ‘key innovation’ triggering species diversification in lizards by facilitating their access to the ecological opportunity offered by otherwise inaccessible cold environments [12], such as high-elevation areas emerging following the uplift of mountain systems. The functional dependence of viviparous species on such cold and highly fluctuating thermal environments [78–80] has been suggested to operate as a limitation for cold-climate adapted species to expand from cold to warm environments in lineages in which reverse transitions from viviparity to oviparity are unlikely to happen [12, 80]. Recent phylogenetic analyses suggest that this is the case for the *Liolaemus* genus (but see [81] for cases of squamates where reversals have been suggested to occur), which has been shown to have an oviparous ancestor and where the predominant tendency is for oviparity-viviparity transitions in multiple independent events, while only one weakly supported case may have experienced a reversal to oviparity from viviparity [12]. *Phymaturus* is a cold climate lineage where, as indicated above, only viviparous parity mode is known. This strong contrast in the degree of both ecological and spatial diversification between *Phymaturus* and *Liolaemus* is consistent with the hypothesis that viviparous parity mode is likely to be predominantly irreversible (at least within this lizard family). The major potential implication for the diversification of *Phymaturus* species is that this lineage is likely to have remained ‘trapped’ within the cold-climates they originated in as a viviparous radiation. Given that *Phymaturus* seem to have been unable to adaptively deviate from the ancestral niche, their adaptive restrictions are likely to explain their extreme contrast in terms of species-richness with the hyper-diversity found in *Liolaemus*, which have extensively radiated across all possible environments [18, 82–84]. These findings relate to the question whether some forms of speciation (e.g., sympatric versus allopatric) are likely to differ in rates as a function of the opportunities for divergence of species into new lineages. For example, while the adaptive potential of *Liolaemus* has allowed them to invade a great range of climates and of microhabitats within each of them, which is reflected by their exceptional species richness, the phylogenetic inertia pulling ecological or life history diversification of *Phymaturus* around the very basal ancestor of the genus has probably been responsible for their comparatively much lower diversity, given the reduced opportunities to proliferate following the ancestral niche.

In addition, their predominant preference for rocky microhabitats [85] further compounds their dispersal by limiting the amount of viable habitats available. Therefore, this combination of factors restricts their opportunities to geographically radiate in environments other than rocky areas in the Andes and Patagonia, where both climate and habitat structures offer the conditions demanded by their strong tendency to retain their ancestral niche. What intrinsic (e.g., genomic) factors have precluded the potential for *Phymaturus* to exploit a wider range of microhabitats (even within Andean-Patagonian regions, in contrast with their sister *Liolaemus*, which have extensively radiated both geographically and ecologically) remains an open question with intriguing implications.

### Niche lability and the potential for assemblage evolution

A consequence of the lack of niche differentiation within the *Phymaturus* genus is that species of this clade are unlikely to efficiently coexist in sympatry [13], as predicted by the competitive exclusion principle [7, 86, 87]. This principle suggests that natural selection emerges when species of a lineage directly engage in conflict as a result of their occupation of overlapping areas of the niche space, which promotes ecological character displacement and thus reciprocal adaptive departures in ecologically relevant traits to reduce the intensity of competition between them. The prevalent adaptive constraints observed in *Phymaturus* are therefore likely to explain their restricted and reciprocally isolated geographic ranges, resulting in a clade of predominantly allopatric species (see Additional File 7) [13]. In fact, some exceptions to this tendency have been found. For example, the species *P. roigorum* and *P. payuniae*, whose coexistence is facilitated through some incipient differences in their use of the same habitat [37, 38]. While *P. payuniae* is typically found on two rock types and actively avoids basaltic rocks when basking, *P. roigorum* is found on many types of rocks and has a wider thermal tolerance [38]. Also, *P. payuniae* has been observed to be the better thermoregulator of the two species [38] and avoids overheating through shorter basking periods and utilising shade more frequently than *P. roigorum* [37]. This case illustrates minor niche differentiation potentially occurring when two *Phymaturus* species come into contact, supporting the prediction that ecological interference among ecologically similar species promotes natural selection for character displacement in traits that engage in direct ecological conflict [2, 88]. While variation in preferred body temperatures have also been observed among a number *Phymaturus* species, their thermal biology is nevertheless thought to be evolutionary constrained [89]. In fact, temperature is known to significantly impact the physiology, micro-habitat availability and spatial distribution of species of the *Liolaemus* genus [90] and of ectotherms in general [91–93].

### The evolution of body size disparity

Our analyses revealed lower relative body size disparity than expected by chance during the first half of the *Phymaturus* history (until ~10Mya), and thus, the limited variation in this trait remained restricted within clades independently with no overlap in morphospace. The surface-based analyses reinforce this finding by showing that only two major transitions in body size are likely to have taken place, one leading to the *palluma* (Andean; with one species that did not undergo this shift) clade, and the other to the *patagonicus* (Patagonian; with one species that recently shifted significantly; see Additional Files 5 and 6) clade. After this period, body size disparity increased substantially, which can be interpreted by higher accumulation of species exposed to a broader range of environmental conditions across the Andes and Patagonia. These climatic gradients are likely to have driven some degree of local adaptation in body size, without having an effect on the main dimensions of the ancestral niche that remained consistently unchanged among newly evolving species (i.e., parity mode, herbivory, use of rocky microhabitats, extremely low fecundity, among others). This transition from extremely low body size disparity recovered for the first half of the *Phymaturus* history, to higher relative disparity resulted in increased overlap of body size plans in the morphospace which, however, is not associated with spatial overlap among species with similar body size plans. In fact, the predominantly allopatric distribution of *Phymaturus* species (see Additional File 7) [33, 35] is likely to have operated as the fundamental factor facilitating the retention of ancestral body size plans given the lack of ecological interference among species, and thus, contributes to the evolutionary stability of body size disparity among species within both major clades.

## Conclusions

Our study reveals a striking case of non-adaptive radiation in the cold-climate lizard genus *Phymaturus*. The genus has experienced a slow accumulation of species early on in their evolutionary history followed by accelerated diversification in the later part. It is likely that the strict habitat requirements of the genus, coupled with the suggested constraints for dispersal across climatic zones imposed by a strictly viviparous reproduction, have limited opportunities for radiations of these reptiles into novel environments, which could also explain their considerably lower diversity of species compared to *Liolaemus*. Body size diversification conforms to a two-peak only model of stabilising selection operating as a strong influence on the maintenance of body sizes within the genus. These two adaptive peaks show minor divergence between certain species supporting conventional theory that species adapt to local differences in climate and habitat during non-adaptive radiations. Species of the Andean dwelling *palluma* group, inhabiting colder climatic conditions at higher altitudes, have evolved larger body sizes as shown by the larger 96.5 mm adaptive peak. The pattern of larger bodied species occupying colder environmental temperatures is the fundamental prediction of Bergmann’s rule, which, despite being consistently rejected in the sister genus *Liolaemus* [20, 64, 94], is a question yet to be statistically analysed in *Phymaturus*. The explicit testing of niche-overlap within the genus, in conjunction with the findings presented here, is needed before *Phymaturus* can be established as an example of a non-adaptive radiation as is often hypothesised. The contrasts between the macroevolutionary dynamics observed in *Phymaturus*, compared to its sister clade *Liolaemus*, open unique opportunities to investigate the factors underlying the extraordinary asymmetries that evolve in nature.

## Additional files

Additional File 1: Accession numbers for all *Phymaturus* and *Liolaemus* species used to create the phylogenetic tree. All sequences downloaded from the Genbank database (http://www.ncbi.nlm.nih.gov/genbank). (DOCX 21 kb)

Additional File 2: NEXUS file of the original phylogenetic tree used for analyses in this paper. (TXT 5 kb)

Additional File 3: Phylogeny of 58 species of *Phymaturus* with node support values. Support values are given to two decimal places to the left of the respective node and above the preceding branch. Those values with posterior probabilities > 0.95 are given in bold. The scale bar represents a branch length of 5 million years (TIFF 323 kb)

Additional File 4: Snout-vent length of all *Phymaturus* species used in the body size analysis (DOCX 14 kb)

Additional File 5: Reconstructed phylogeny showing convergences of body size peaks in *Phymaturus.* Red branch tips indicate species subject to the smaller body size regime at 86.4 mm (4.46 ln-transformed), also indicated by the open circles labelled ‘2’ and ‘3’. Black branch tips represent those species subject to the larger body size regime at 96.5 mm (4.57 ln-transformed) (PNG 11 kb)

Additional File 6: Distribution of regimes of SVL in *Phymaturus*. Circles indicate the same SVL regimes as additional file 4 and are coloured accordingly. The larger circles represent the mean of each SVL regime with smaller circles showing the distribution of species within each regime. (PNG 21 kb)

Additional File 7: Distribution map of *Phymaturus* species across Chile and Argentina. Members of the clade are assigned a unique symbol illustrating their allopatric distributions. Altitude is given in metres above sea level. (TIFF 378 kb)

## Abbreviations

AIC: Akaike Information Criterion
BM: Brownian motion model
crBD: constant rate birth-death model
DDE + E: density-dependent exponential model
DDL + E: density-dependent logistic model
DTT: disparity through time
EB: early burst
HMM: hidden Markov model
LTT: lineage through time plot
MCCR: Monte Carlo Constant Rate
MDI: morphological disparity index
Mya: million years ago
OU: Ornstein Uhlenbeck model
PNC: phylogenetic niche conservatism
SVL: snout-vent length
