# Supplementary material for: Macroevolutionary diversification with limited niche disparity in a species-rich lineage of cold-climate lizards
Source: BMC Evol Biol. 2018 Feb 6;18:16. doi: 10.1186/s12862-018-1133-1 (PMC5801843; doi:10.1186/s12862-018-1133-1)
Supplement: Supplementary file 1 — Accession numbers for all Phymaturus and Liolaemus species used to create the phylogenetic tree. All sequences downloaded from the Genbank database (http://www.ncbi.nlm.nih.gov/genbank). (DOCX 21 kb) [file 12862_2018_1133_MOESM1_ESM.docx]

**Additional File 1:** Accession numbers for all *Phymaturus* and *Liolaemus* species used to create the phylogenetic tree. All sequences downloaded from the Genbank database (<http://www.ncbi.nlm.nih.gov/genbank>).

| Species | **Genes and GenBank Accession No.** | | |
| --- | --- | --- | --- |
|  | 12S | Cytb | C-mos |
| *Liolaemus azarai* | KF969006 | JN614931 | KF968634 |
| *Liolaemus bibronii* | JN410401 | JN410551 | KF968637 |
| *Liolaemus boulengeri* | KF969197 | KF968990 | KF968814 |
| *Liolaemus cuyanus* | DQ237586 | DQ237490 | KF968654 |
| *Liolaemus chacoensis* | DQ237590 | JN614927 | JN614965 |
| *Liolaemus chehuachekenk* | KF969022 | JN614925 | KF968651 |
| *Liolaemus darwinii* | JF272804 | KC150517 | KF968657 |
| *Liolaemus donosobarrosi* | KF969030 | JN614924 | KF968659 |
| *Liolaemus elongatus* | AY173922 | AY173855 | JN614959 |
| *Liolaemus famatinae* | DQ237469 | DQ237485 | KF968666 |
| *Liolaemus fitzingerii* | DQ237569 | DQ237488 | KF968667 |
| *Liolaemus kingii* | AY367835 | DQ237489 | KF968681 |
| *Liolaemus magellanicus* | JF272812 | JF272779 | KF968694 |
| *Liolaemus multimaculatus* | KF969088 | KF968892 | KF968711 |
| *Liolaemus pseudoanomalus* | KF969094 | AY367796 | KF968717 |
| *Liolaemus rothi* | KF969198 | KF968991 | KF968815 |
| *Liolaemus telsen* | KF969182 | KF968995 | KF968818 |
| *Liolaemus vallecurensis* | AY367838 | AY367808 | KF968785 |
| *Liolaemus wiegmannii* | DQ237471 | DQ237487 | KF968794 |
| *Phymaturus aguedae* | KT203835 | KT203826 | KT203818 |
| *Phymaturus antofagastensis* | KT203842 | KT203828 | KT203824 |
| *Phymaturus bibronii* | KT203840 | KT203829 | KT203822 |
| *Phymaturus cacivoi* | JX969106 | JX969055 | JX969559 |
| *Phymaturus calcogaster* | JX969081 | JX969030 | JX969535 |
| *Phymaturus camilae* | JX969110 | JX969059 | JX969563 |
| *Phymaturus ceii* | JX969082 | JX969031 | JX969536 |
| *Phymaturus damasense* | KT203843 | KT203830 | KT203825 |
| *Phymaturus delheyi* | JX969098 | JX969047 | JX969551 |
| *Phymaturus denotatus* | KT203838 | KT203833 | KT203820 |
| *Phymaturus dorsimaculatus* | JF272814 | JF272781 | JX969518 |
| *Phymaturus etheridgei* | JX969080 | JX969029 | JX969534 |
| *Phymaturus excelsus* | JX969083 | JX969032 | JX969537 |
| *Phymaturus extrilidus* | JX969070 | JX969019 | JX969527 |
| *Phymaturus felixi* | JX969095 | JX969044 | JX969549 |
| *Phymaturus indistinctus* | JX969084 | JX969033 | JX969538 |
| *Phymaturus laurenti* | JX969060 | JX969009 | JX969517 |
| *Phymaturus mallimaccii* | JX969062 | JX969011 | JX969519 |
| *Phymaturus manuelae* | JX969085 | JX969034 | JX969539 |
| *Phymaturus maulense* | JX969071 | JX969045 |  |
| *Phymaturus nevadoi* | JX969086 | JX969035 | JX969540 |
| *Phymaturus palluma* | AY662050 | KT203834 | JX969520 |
| *Phymaturus paihuanense* | KT203841 | KT203832 | KT203823 |
| *Phymaturus patagonicus* | JX969087 | JX969036 | JX969541 |
| *Phymaturus payuniae* | JX969088 | JX969037 | JX969542 |
| *Phymaturus punae* | JX969064 | JX969013 | JX969521 |
| *Phymaturus querque* | JX969069 | JX969018 | JX969526 |
| *Phymaturus rahuensis* | JX969104 | JX969053 | JX969557 |
| *Phymaturus roigorum* | JX969065 | JX949099 | JX969522 |
| *Phymaturus sitesi* | JX969100 | JX948830 | JX969553 |
| *Phymaturus somuncurensis* | JX969089 | JX969038 | JX969543 |
| *Phymaturus spectabilis* | JX969090 | JX969039 | JX969544 |
| *Phymaturus spurcus* | JX969091 | JX969040 | JX969545 |
| *Phymaturus tenebrosus* | JX969092 | JX969041 | JX969546 |
| *Phymaturus* *tromen* | JX969068 | JX969017 | JX969525 |
| *Phymaturus verdugo* | JX969066 | JX969015 | JX969523 |
| *Phymaturus videlai* |  | JX949164 |  |
| *Phymaturus vociferator* | JX969067 | JX969016 | JX969524 |
| *Phymaturus williamsi* | KT203837 | KT203827 |  |
| *Phymaturus yachanana* | JX969109 | JX969058 | JX969562 |
| *Phymaturus zapalensis* | JX969093 | JX969042 | JX969547 |
| *Phymaturus sp. fia* | KT203836 | KT203831 | KT203819 |
| *Phymaturus sp. gua* | KT203844 |  |  |
| *Phymaturus sp. 3* | JX969072 | JX969021 |  |
| *Phymaturus sp. 4* | JX969073 | JX969022 | JX969528 |
| *Phymaturus sp. 5* | JX969074 | JX969023 |  |
| *Phymaturus sp. 6* | JX969075 | JX969024 | JX969529 |
| *Phymaturus sp. 7* | JX969076 | JX969025 | JX969530 |
| *Phymaturus sp. 8* | JX969077 | JX969026 | JX969531 |
| *Phymaturus sp. 9* | JX969078 | JX969027 | JX969532 |
| *Phymaturus sp. 10* | JX969079 | JX969028 | JX969533 |
| *Phymaturus sp. 12* | JX969099 | JX969048 | JX969552 |
| *Phymaturus sp. 13* | JX969101 | JX969050 | JX969554 |
| *Phymaturus sp. 14* | JX969102 | JX969051 | JX969555 |
| *Phymaturus sp. 15* | JX969103 | JX969052 | JX969556 |
| *Phymaturus sp. 17* | JX969105 | JX969054 | JX969558 |
| *Phymaturus sp. 20* | JX969108 | JX969057 | JX969561 |
| *Phymaturus sp. 22* | JX969097 | JX969046. | JX969550 |
