# Supplementary figures and images for: Macroevolutionary diversification with limited niche disparity in a species-rich lineage of cold-climate lizards
Source: BMC Evol Biol. 2018 Feb 6;18:16. doi: 10.1186/s12862-018-1133-1 (PMC5801843; doi:10.1186/s12862-018-1133-1)

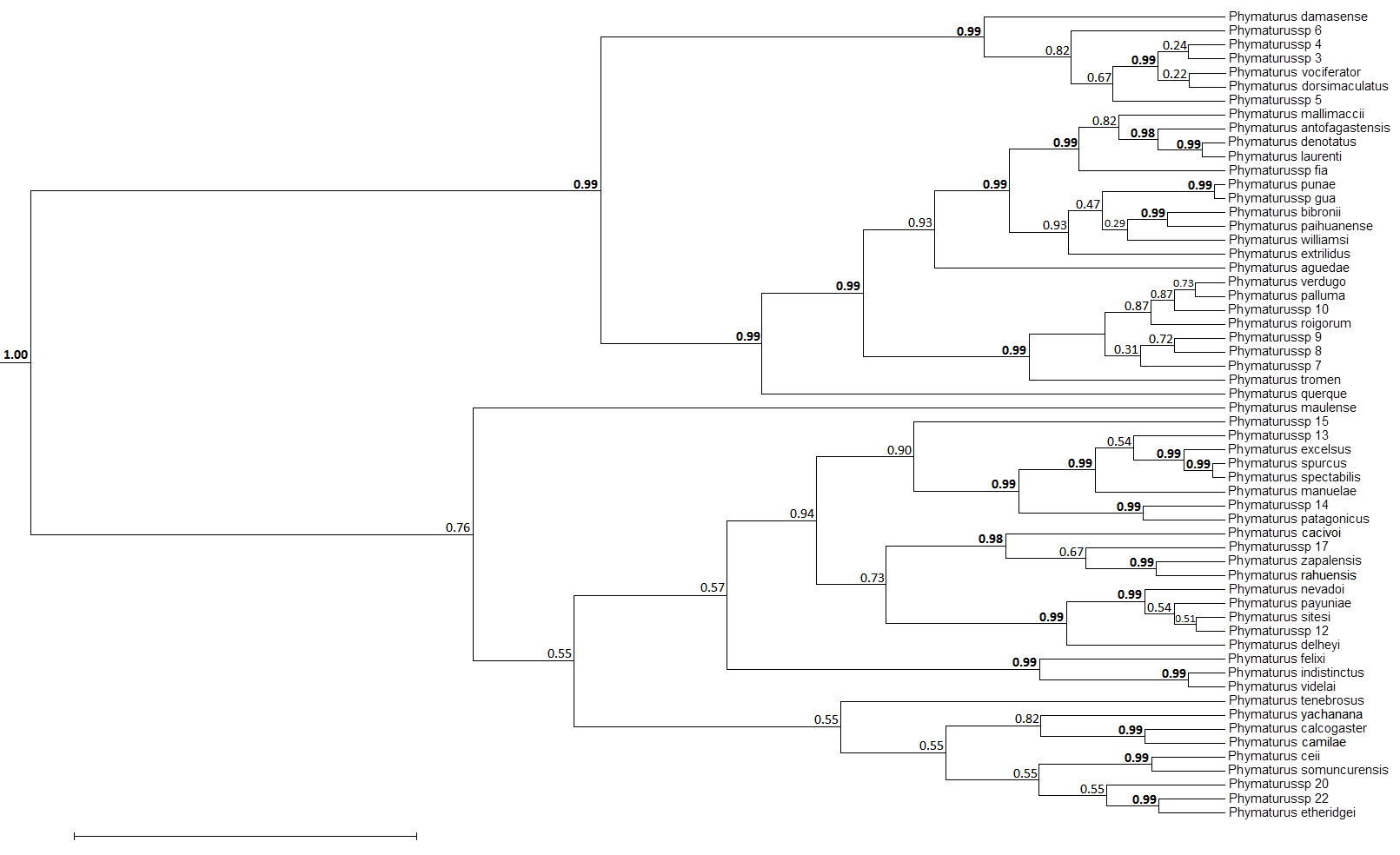

Supplement: Supplementary file 3 — Phylogeny of 58 species of Phymaturus with node support values. Support values are given to two decimal places to the left of the respective node and above the preceding branch. Those values with posterior probabilities > 0.95 are given in bold. The scale bar represents a branch length of 5 million years (TIFF 323 kb) [file 12862_2018_1133_MOESM3_ESM.tif]

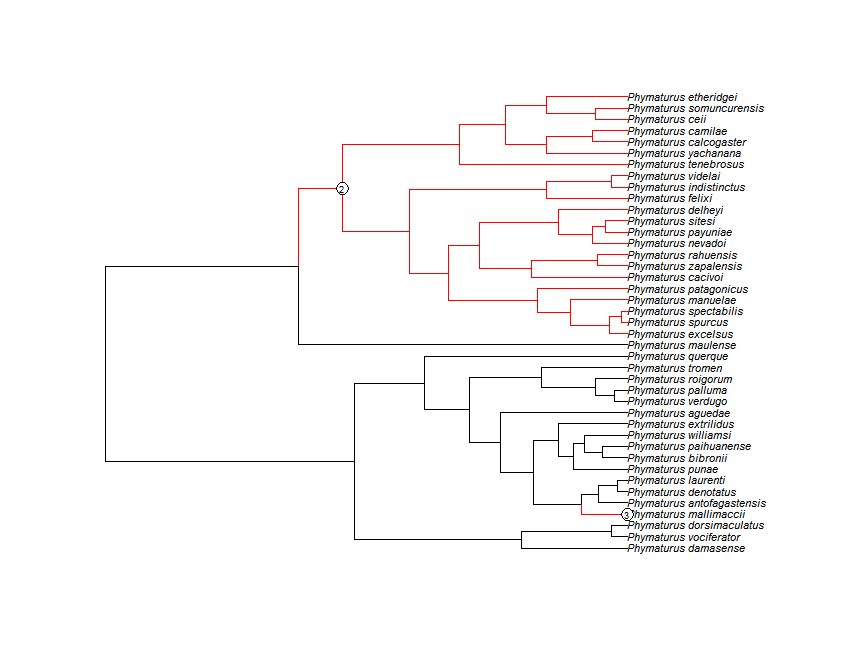

Supplement: Supplementary file 5 — Reconstructed phylogeny showing convergences of body size peaks in Phymaturus. Red branch tips indicate species subject to the smaller body size regime at 86.4 mm (4.46 ln-transformed), also indicated by the open circles labelled ‘2’ and ‘3’. Black branch tips represent those species subject to the larger body size regime at 96.5 mm (4.57 ln-transformed) (PNG 11 kb) [file 12862_2018_1133_MOESM5_ESM.png]

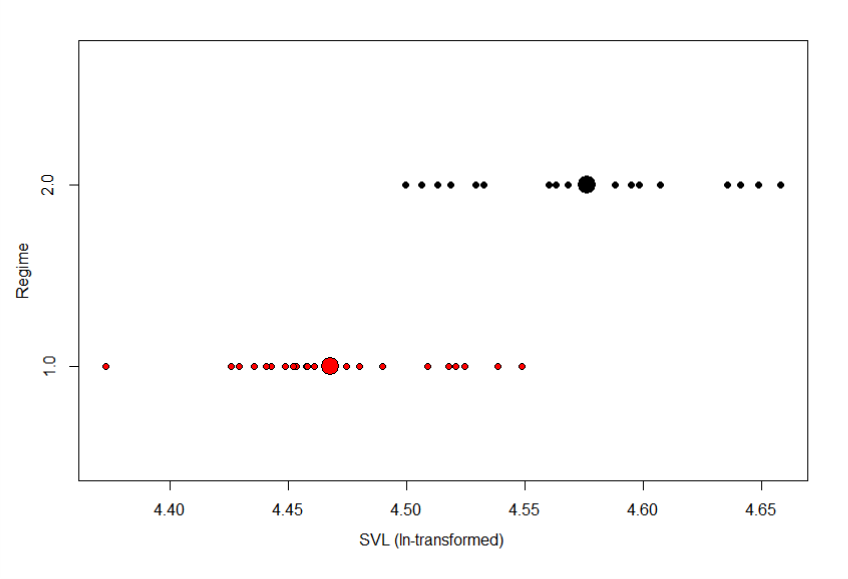

Supplement: Supplementary file 6 — Distribution of regimes of SVL in Phymaturus. Circles indicate the same SVL regimes as additional file 4 and are coloured accordingly. The larger circles represent the mean of each SVL regime with smaller circles showing the distribution of species within each regime. (PNG 21 kb) [file 12862_2018_1133_MOESM6_ESM.png]

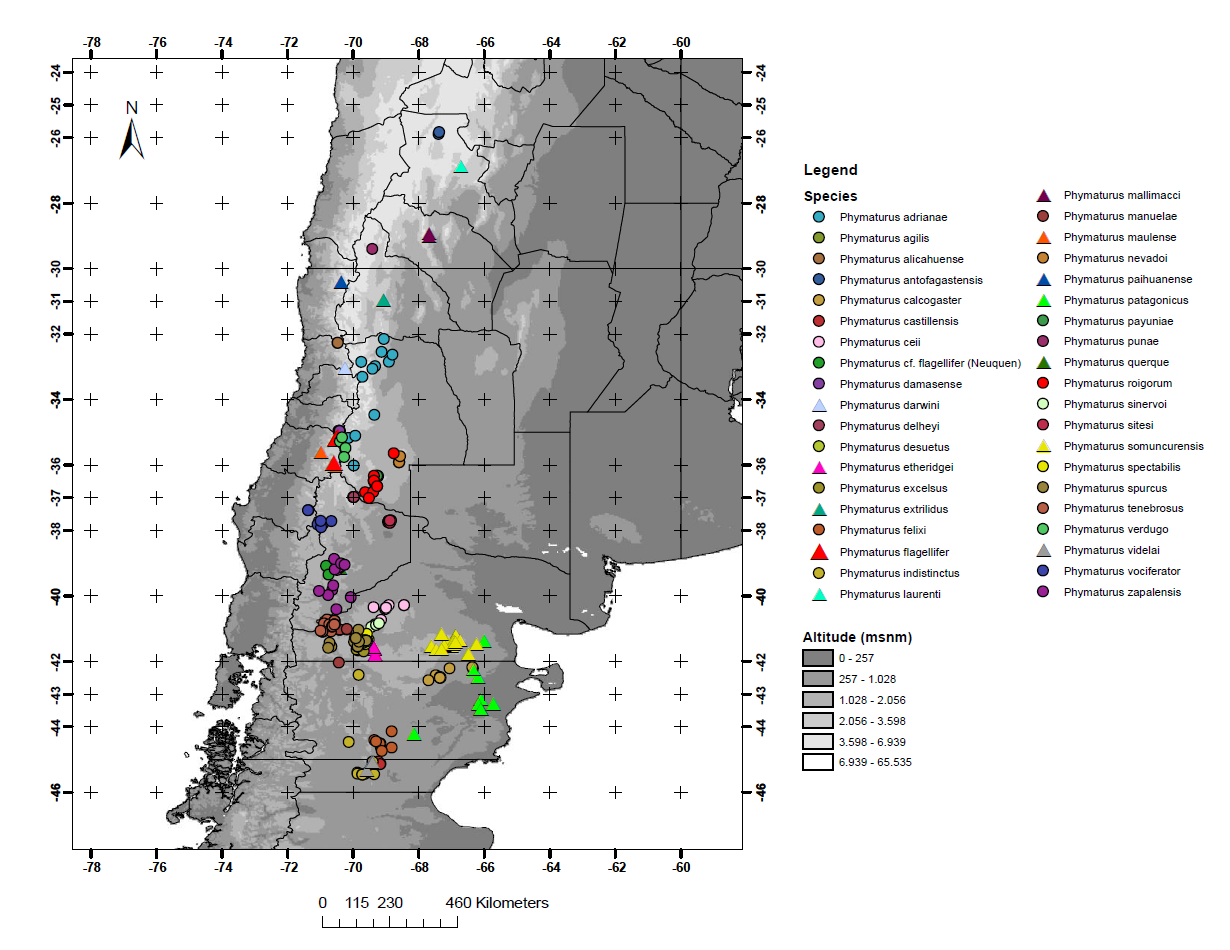

Supplement: Supplementary file 7 — Distribution map of Phymaturus species across Chile and Argentina. Members of the clade are assigned a unique symbol illustrating their allopatric distributions. Altitude is given in metres above sea level. (TIFF 378 kb) [file 12862_2018_1133_MOESM7_ESM.tif]
