# Supplementary material for: Macroevolutionary diversification with limited niche disparity in a species-rich lineage of cold-climate lizards
Source: BMC Evol Biol. 2018 Feb 6;18:16. doi: 10.1186/s12862-018-1133-1 (PMC5801843; doi:10.1186/s12862-018-1133-1)
Supplement: Supplementary file 4 — Snout-vent length of all Phymaturus species used in the body size analysis (DOCX 14 kb) [file 12862_2018_1133_MOESM4_ESM.docx]

**Additional File 4:** Snout-vent length (SVL, in mm) data of the *Phymaturus* species used in this study.

| Species | Body size Data | |
| --- | --- | --- |
|  | *N* | SVL |
| *Phymaturus aguedae*  *Phymaturus antofagastensis*  *Phymaturus bibronii*  *Phymaturus cacivoi*  *Phymaturus calcogaster*  *Phymaturus camilae*  *Phymaturus ceii*  *Phymaturus damasense*  *Phymaturus delheyi*  *Phymaturus denotatus*  *Phymaturus dorsimaculatus*  *Phymaturus etheridgei*  *Phymaturus excelsus*  *Phymaturus extrilidus*  *Phymaturus felixi*  *Phymaturus indistinctus*  *Phymaturus laurenti*  *Phymaturus mallimaccii*  *Phymaturus manuelae*  *Phymaturus maulense*  *Phymaturus nevadoi*  *Phymaturus paihuanense*  *Phymaturus palluma*  *Phymaturus patagonicus*  *Phymaturus payuniae*  *Phymaturus punae*  *Phymaturus querque*  *Phymaturus rahuensis*  *Phymaturus roigorum*  *Phymaturus sitesi*  *Phymaturus somuncurensis*  *Phymaturus spectabilis*  *Phymaturus spurcus*  *Phymaturus tenebrosus*  *Phymaturus tromen*  *Phymaturus verdugo*  *Phymaturus videlai*  *Phymaturus vociferator*  *Phymaturus williamsi*  *Phymaturus yachanana*  *Phymaturus zapalensis* | –^1^  40  –^1^  –^1^  39  17  49  8  –^1^  –^1^  –^1^  26  46  6  30  23  9  11  18  14  17  12  96  76  74  41  8  –^1^  84  11  59  137  34  68  –^1^  16  17  52  –^1^  –^1^  74 | 89.9  91.7  103.1  91.9  83.6  92.2  86.3  104.4  89.1  105.5  91.2  84.4  84.8  98.9  85.5  85.8  90.6  85.0  90.8  92.7  88.2  99.3  95.9  86.6  85.9  96.4  98.3  86.3  100.2  85.9  91.7  83.9  87.8  93.6  95.6  103.7  79.3  92.9  100.2  94.5  83.6 |

^1^Data taken from the original sources where the species were first described
